# miR-103/miR-195/miR-15b Regulate SALL4 and Inhibit Proliferation and Migration in Glioma

**DOI:** 10.3390/molecules23112938

**Published:** 2018-11-10

**Authors:** Li-Ping Chen, Na-Na Zhang, Xue-Qing Ren, Jie He, Yu Li

**Affiliations:** School of Life Science and Technology, Harbin Institute of Technology, Harbin 150080, China; chenlp0536@163.com (L.-P.C.); silvernaz@126.com (N.-N.Z.); renxueqing3089@163.com (X.-Q.R.)

**Keywords:** miR-103, miR-195, miR-15b, SALL4, glioma

## Abstract

Glioma is the common highly malignant primary brain tumor. However, the molecular pathways that result in the pathogenesis of glioma remain elusive. In this study, we found that microRNA-103 (miR-103), microRNA-195 (miR-195), or microRNA-15b (miR-15b), which all have the same 5′ “seed” miRNA portion and share common binding sites in the SALL4 3′-untranslated region (UTR), were downregulated in glioma tissues and cell lines. These miRNAs suppressed glioma cell proliferation, migration, and invasion, induced cell apoptosis, and decreased the level of the SALL4 protein, but not that of SALL4 mRNA, which was identified as a direct target of all three miRNAs. The caspase-3/7 activity expression in U251 cells overexpressing these miRNAs was rescued during SALL4 upregulation. An obvious inverse correlation was observed between SALL4 and miR-103 or miR-195 expression levels in clinical glioma samples. Moreover, enforced expression of SALL4 stimulated cell proliferation, migration, and invasion. In conclusion, these data suggest that miR-103, miR-195, and miR-15b post-transcriptionally downregulated the expression of SALL4 and suppressed glioma cell growth, migration, and invasion, and increased cell apoptosis. These results provide a potential therapeutic target that may downregulate SALL4 in glioma.

## 1. Introduction

Glioma is the common highly malignant primary brain tumor, and despite recent advances in the currently available therapeutic interventions, the median time of survival is less than 16 months after diagnosis [1]. Understanding the molecular pathways that lead to glioma is crucial for identifying new targets for anticancer drug development.

SALL4, a zinc-finger transcription factor, is a member of the *SALL* gene family, and is located on chromosome 20. It was first cloned based on its sequence homology with *Drosophila* spalt [2,3]. Human SALL4 plays a pivotal role in maintaining the pluripotency and self-renewal characteristics of embryonic and hematopoietic stem cells [4,5]. However, growing evidence suggests that SALL4 is also associated with carcinogenesis and metastasis. SALL4 expression has been found in various types of tumors, including human acute myeloid leukemia, as well as lung, liver, gastric, and endometrial cancers [6,7,8,9,10]. Previously, we also demonstrated that SALL4 is critical for gliomagenesis, and that the upregulation of miR-107 suppresses glioma cell growth through direct targeting of SALL4 [11]. Other groups have also indicated that miRNA-98, miRNA-33b, and miRNA-219 play tumor-suppressive roles in cancer by targeting SALL4 [12,13,14,15]. However, the mechanisms controlling glioma cell proliferation, migration, and invasion through miRNAs targeting SALL4 remain relatively unknown.

miRNAs are small noncoding RNAs comprised of approximately 22 nucleotides that bind to the 3′UTR region of target mRNAs, and act as post-transcriptional regulators of mRNA expression via destabilization or translational repression [16]. Through this mechanism, miRNAs play critical roles in pathogenesis and cancer progression [17,18]. Several prior studies indicated that miR-103, miR-195, and miR-15b make functional contributions to tumorigenesis. For example, miR-103 can promote or inhibit cellular proliferation by targeting tumor suppressors or oncogenes [19,20,21,22]. Kfir-Erenfeld et al. demonstrated that miR-103 inhibits leukemia cellular proliferation by reducing the expression of cyclin dependent kinase (CDK2) and its cyclin E1 target [20]. However, Yu et al. suggested that miR-103 downregulates the expression of the tumor suppressor TIMP-3, and stimulates growth in endometrial cancer cell lines [22]. miR-195 has been found to play a tumor-suppressor role in human glioblastoma U87 cells by targeting signaling pathways involved in cellular proliferation [23]. In contrast, Moser et al. reported increased levels of miR-195 in U87 cells, suggesting that miR-195 acts as an oncogene [24]. MacLean et al. showed that miR-15b inhibits cellular proliferation by regulating WNT7A in ovarian cancer cells [25]. By contrast, Chen et al. revealed that miR-15b facilitates tumorigenicity by targeting RECK in prostate cancer cells [26]. The above studies indicate that the exact functions of miR-103, miR-195, and miR-15b are highly cell type-dependent, and still controversial. Thus, there is a greater need to explore the role and clinical significance of these three miRNAs in gliomagenesis.

In the present study, a bioinformatics analysis is used to identify SALL4 as a putative target of miR-103, miR-195, and miR-15b (Figure S1). Based on a bioinformatics prediction, these three miRNAs are selected for a more detailed study to test whether they affect gliomagenesis and invasion, and how this occurs. Our data show that the three tested miRNAs are downregulated in primary glioma tissues and cell lines. They inhibit carcinogenesis, migration, and invasion, and significantly induce apoptosis in glioma cells. Their upregulation or inhibition decreases or increases SALL4 protein expression, and they directly target SALL4 in glioma. Rescue experiments with the caspase-3/7 activity assay show that SALL4 overexpression reverses the apoptosis-inductive effects of the three miRNAs. Moreover, the relationship between the expression of miR-103 or miR-195, and the SALL4 level, has an inverse correlation in glioma patients. SALL4 overexpression elevates cell growth, migration, and invasion. In conclusion, our work reveals that miR-103, miR-195, and miR-15b has a suppressive effect on cellular proliferation and invasion in glioma. Therefore, the miRNAs/SALL4 axis may become a potential therapeutic target for glioma.

## 2. Results

### 2.1. Downregulation of miR-103, miR-195, and miR-15b in Glioma Clinical Samples and Glioma Cell Lines

**Figure 1:**
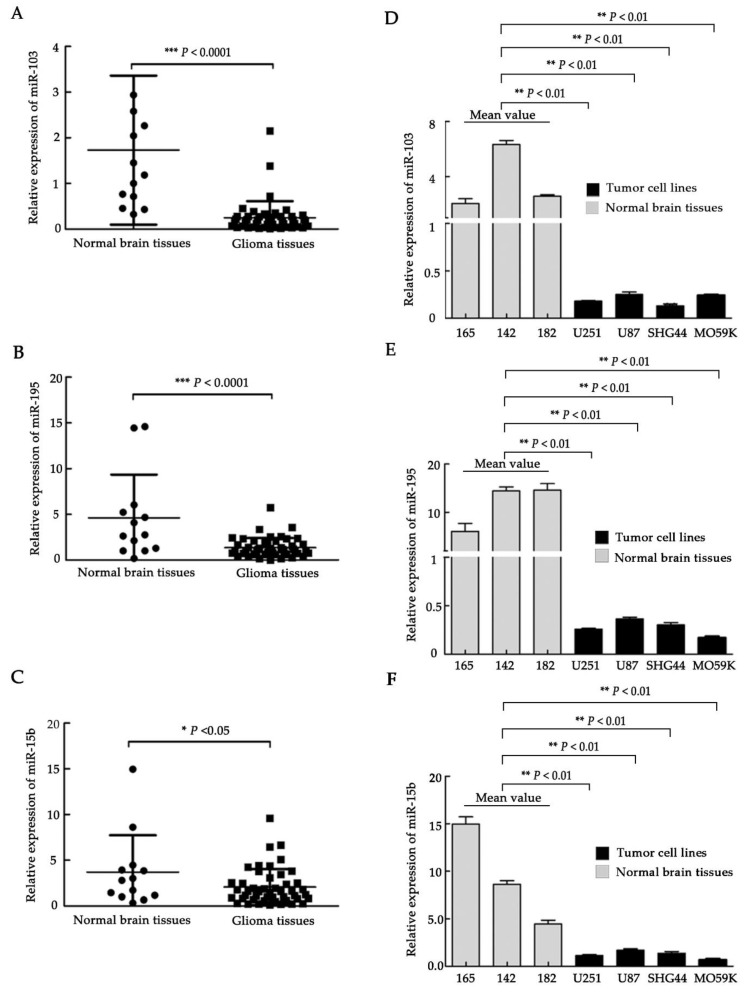
miR-103, miR-195, and miR-15b expression in human glioma tissues and cell lines. (**A**–**C**) miR-103, miR-195, and miR-15b expression was assessed by qRT-PCR in glioma tissues (*n* = 47) and normal brain tissues (*n* = 13). (**D**–**F**) The relative levels of miR-103, miR-195, and miR-15b in human glioma cell lines. 165, 142, and 182 are three representative normal human brain tissues that were chosen randomly from 13 human normal brain tissues used in Figure 1A–C. Values (means ± SD, *n* = 3) are shown. *, *p <*0.05; **, *p <*0.01; ***, *p <*0.001.

To elucidate the relationship between the occurrence of glioma and the relative expression of miR-103, miR-195, and miR-15b, which contain the same 5′ “seed” portion, we compared 47 clinical samples (WHO I, *n* = 12; WHO II, *n* = 13; WHO III, *n* = 12; WHO IV, *n* = 10) with 13 normal brain tissue samples by using qRT-PCR (Figures S1 and S2, Figure 1A–C). Compared to normal brain tissues, the expression of miR-103, miR-195, and miR-15b was statistically significantly downregulated in tumor tissues (Figure 1A–C, *p <*0.0001, *p <*0.0001, *p <*0.05, respectively). We also examined the expression of the three miRNAs in several glioma cell lines, including U251, U87, MO59K, and SHG44. Normal brain tissues were used as a control. Our data showed that the expression levels of the tested miRNAs in the four glioma cell lines were significantly lower than in normal brain tissues (Figure 1D–F, *p*< 0.01). 

### 2.2. miR-103, miR-195, and miR-15b Impede Cell Proliferation via Apoptosis

**Figure 2:**
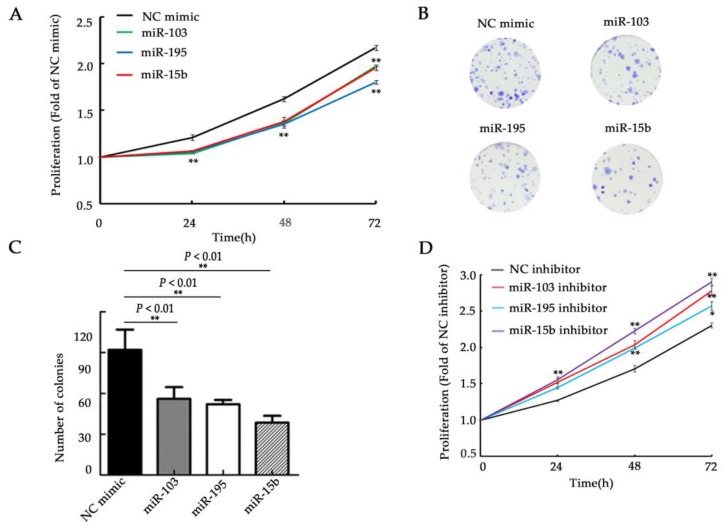

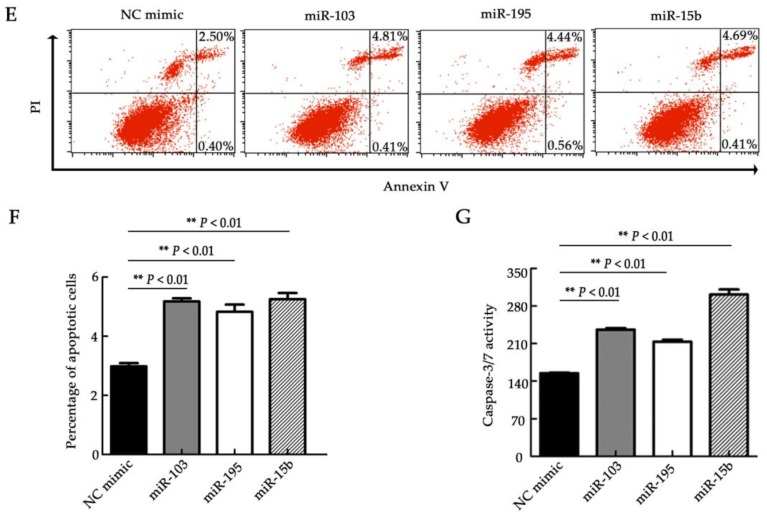
miR-103, miR-195, and miR-15b suppressed cell proliferation and increased apoptosis. (**A**) MTT proliferation assays in U251 cells following transfection with miR-103, miR-195, and miR-15b mimics. (**B**,**C**) Colony formation assays in U251 cells. The colonies were imaged (**B**) and counted (**C**). (**D**) The MTT proliferation analysis was performed in U87 cells after transfection with inhibitors of the three miRNAs. (**E**,**F**) Annexin V-PI staining indicated increased apoptotic cells in U251 cells after overexpression of the three miRNAs. (**E**) Representative flow cytometric analysis of annexin V-PI staining. (**F**) Percentage of apoptotic cells. (**G**) A statistically significant upregulation in caspase-3/7 activity was found in U251 cells transfected with miR-103, miR-195, and miR-15b mimics, compared with the controls. NC mimic: the negative control of the miRNA mimics. NC inhibitor: the negative control of the miRNA inhibitors. Means ± SD (*n* = 3) are shown. *, *p <* 0.05; **, *p <* 0.01 (*t*-test).

To assess the cell biology functions of miR-103, miR-195, and miR-15b in glioma, their expression levels were verified by using qRT-PCR with a miRNA mimic into U251 cells 48 hours after transfection. The transfection group of miR-103, miR-195, and miR-15b showed a dramatic increase in the expression of corresponding miRNAs, compared with negative control cells (Figure S3, *p <* 0.001). We then tested the proliferation kinetics following miRNA transfection by the MTT assay. Overexpression of miR-103, miR-195, and miR-15b significantly inhibited growth to different degrees when compared to the negative control (Figure 2A, *p <* 0.01). Cell proliferation was also assessed with colony formation assays. The results revealed that treatment with the three miRNAs significantly reduced the colony number compared with the U251 control (Figure 2B,C, *p <* 0.01). U87 cells, which have a relatively high express of miR-103, miR-195, and miR-15b in the four glioma cell lines (U87, U251, SHG44, and MO59K), were treated with inhibitors to determine whether they are involved in glioma cancer cell growth. Quantitative reverse transcript-PCR (qRT-PCR) results indicated that the inhibitors of miR-103, miR-195, or miR-15b significantly reduced the expression of the corresponding miRNA after single transfection in U87 cells compared with the negative control (Figure S4, *p <* 0.001). Additionally, the MTT assay demonstrated increased proliferation of U87 cells to different extents compared with the control group (Figure 2D, *p <* 0.01). To further examine whether the block in cell proliferation is due to a cell cycle arrest, cell cycle progression was analyzed by propidium iodide staining and flow cytometric analysis. However, we did not observe any change in the cell cycle (data not shown). The decreased cell proliferation could be due to increased cell death. To explore this possibility, annexin V and PI double staining was performed. The results revealed that single transfection of miR-103, miR-195, and miR-15b all led to a significant increase in the mean percentage of apoptotic cells relative to the negative control (Figure 2E,F, *p <* 0.01). We also observed that overexpression of the three miRNAs resulted in a significant increase in caspase-3/7 activity compared with the control group (Figure 2G, *p <* 0.01). Taken together, these data show that miR-103, miR-195, and miR-15b suppress cell proliferation and induce cell apoptosis.

### 2.3. miR-103, miR-195, and miR-15b Suppress Cell Migration and Invasion

**Figure 3:**
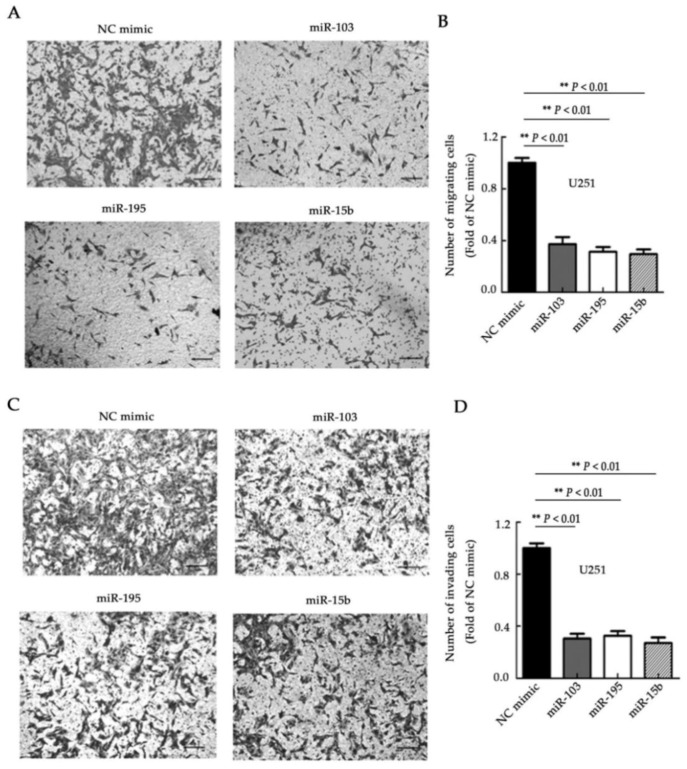
miR-103, miR-195, and miR-15b inhibit cell migration and invasion. (**A**,**B**) Cell migration assays were performed in U251 cells after transfection with the three miRNAs. The migrating cells were imaged (**A**) and counted (**B**) under representative microscopic fields. (**C**,**D**) U251 cells were subjected to invasion assays after transfection with miR-103, miR-195, and miR-15b. The invasive cells were imaged (**C**) and counted (**D**) under representative microscopic fields. NC mimic: negative controls of the mimics of the three miRNAs. Bar =50μm. Means ± SD (*n* = 3) are shown. **, *p <*0.01 (*t*-test).

**Figure 4:**
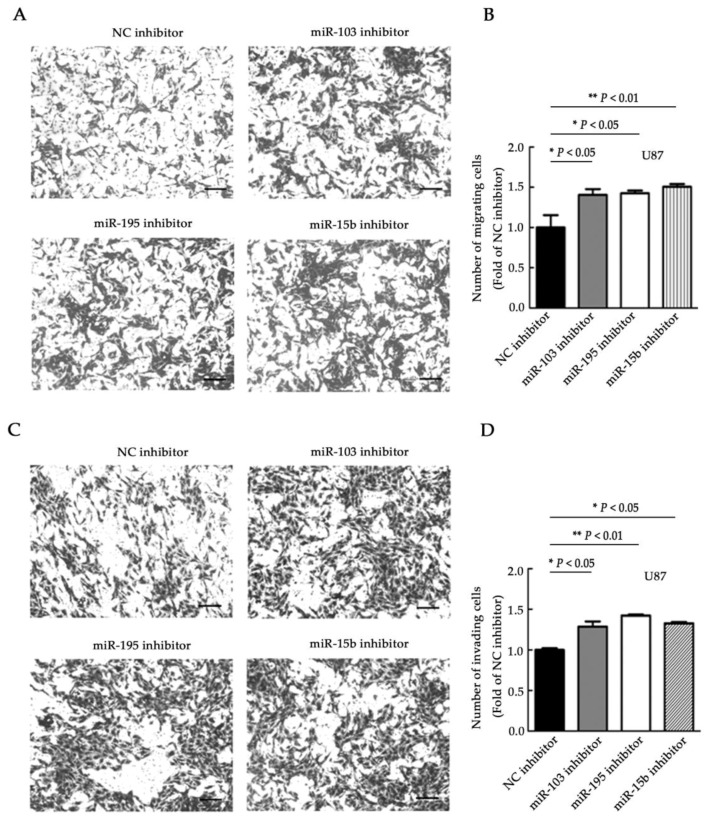
The downregulation of miR-103, miR-195, and miR-15b promotes cell migration and invasion. (**A**,**B**) Cell migration was examined in U87 cells after transfection with miR-103, miR-195, or miR-15b inhibitors. The migrating cells were imaged (**A**) and counted (**B**) under representative microscopic fields. (**C**,**D**) The U87 cells were transfected with the miR-103, miR-195, or miR-15b inhibitors, and cell invasion was examined. The invasive cells were imaged (**C**) and counted (**D**) under representative microscopic fields. NC inhibitor: negative controls of the miRNA inhibitors. Bar = 50μm. Means ± SD (*n* = 3) are shown. *, *p <* 0.05; **, *p <* 0.01 (*t*-test).

To further explore the potential roles of miR-103, miR-195, and miR-15b, transwell assays were utilized to measure the migration and invasion ability of glioma cells. The migratory capacity was significantly reduced in singly transfected cells compared with the control cells (Figure 3A,B, *p <* 0.01). In addition, the invasive capability of U251 cells was significantly suppressed with overexpression of the three miRNAs (Figure 3C,D, *p <* 0.01). To further validate the reductions in cell migration and invasion ability due to these miRNAs, miR-103, miR-195, or miR-15b inhibitors were transfected into U87 cells. When compared to the control group, suppression of the miRNAs markedly increased cell migration and invasion (Figure 4A–D, *p <* 0.05, and *p <* 0.01). These data determined the further processes of the three miRNAs related to the migration and invasion of glioma cancer cells. 

### 2.4. miR-103, miR-195, and miR-15b Directly Target SALL4 3′UTR

**Figure 5:**
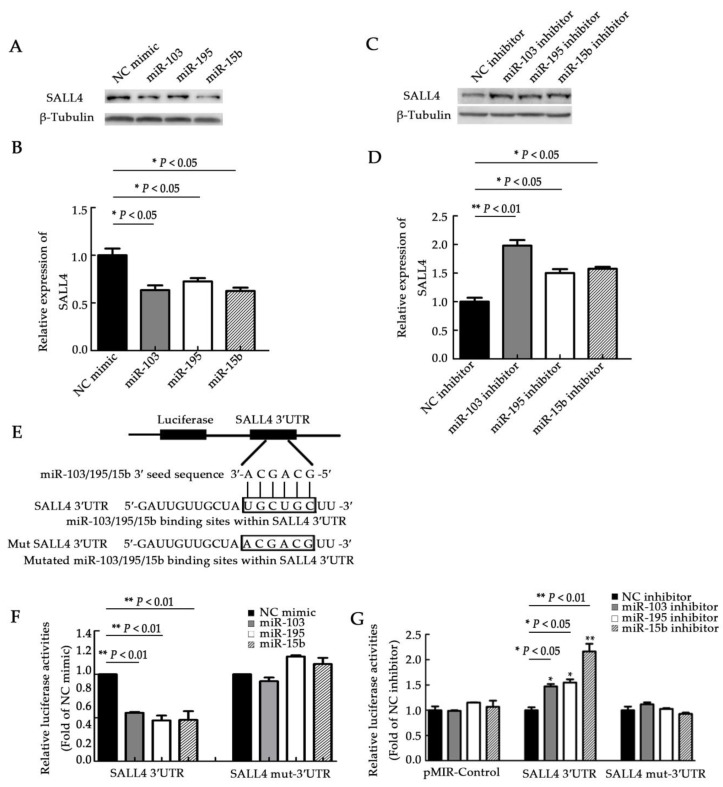
SALL4 is a downstream target of miR-103, miR-195, and miR-15b in glioma cells. (**A**,**B**) Western blot analysis of SALL4 expression. The SALL4 level was significantly reduced in U251 cells after transfection with miR-103, miR-195, and miR-15b mimics. The relative intensity analysis of SALL4/β-tubulin in (**A**) is shown in (**B**). (**C**,**D**) Western blot analysis of SALL4 expression in U87 cells 48 hours after transfection with miR-103, miR-195, and miR-15b inhibitors. The relative intensity analysis of SALL4/β-tubulin of (**C**) is shown in (**D**). (**E**) Diagram of the wild and mutant SALL4-3′UTR sequences. The sites of the seed sequences in miR-103, miR-195, and miR-15b match the 3′UTR of the SALL4 mRNA. (**F**,**G**) Luciferase activity assays were measured in U251 (**F**) and U87 (**G**) cells. Luciferase activity controlled by the 3′UTR of SALL4 was inhibited or promoted by the mimics (**F**) or inhibitors (**G**) of miR-103, miR-195, or miR-15b. NC mimic: negative control of the miRNA mimics. NC inhibitor: negative control of the miRNA inhibitors. Means ± SD (*n* = 3) are shown. *, *p <* 0.05; **, *p <* 0.01 (*t*-test).

Using the TargetScan and miRBase targets, we found that the same seed sequences of miR-103, miR-195, and miR-15b matched the same SALL4 mRNA 3′UTR binding site (Figure S1). The expression of SALL4 mRNA and protein were assessed by qRT-PCR and Western blots after treatment with miR-103, miR-195, and miR-15b mimics or inhibitors, to further confirm the effects of miRNA interactions with this binding site. The qRT-PCR results showed no significant differences in SALL4 mRNA levels between transfections of miR-103, miR-195, or miR-15b, and the control in U251 cells (Figure S5A). Similarly, the transfection of the miRNA inhibitors in U87 cells did not lead to any prominent differences in SALL4 mRNA levels when compared with the control group (Figure S5B). However, the Western blot results revealed that SALL4 protein levels were significantly suppressed after the overexpression of the three miRNAs into U251 cells, and were upregulated after transfection of the miRNA inhibitors into U87 cells. These results were compared with the corresponding control groups (Figure 5A–D, *p <* 0.05, and *p <* 0.01). Additionally, a luciferase reporter assay was performed. Six nucleotides were mutated in the predicted binding sites of miR-103, miR-195, and miR-15b, and both the mutated and wild type 3′UTR of SALL4 were cloned downstream of a luciferase reporter gene. The luciferase activity was assessed in the presence of the *Renilla* luciferase vector and miR-103, miR-195, or miR-15b mimics (Figure 5E,F). Overexpression of the miRNAs significantly decreased the luciferase activity in U251 cells transfected with the wild type 3′UTR of SALL4, but not in cells with a muted type of SALL4-3′UTR (Figure 5F, *p* < 0.01). Conversely, miRNA suppression significantly increased native SALL4-3′UTR luciferase activity in U87 cells (Figure 5G, *p <* 0.05, and *p <* 0.01). This change in activity was not observed in the presence of the mutant SALL4-3′UTR luciferase reporter plasmids (Figure 5G). These results confirm the bioinformatic predictions, which indicate that miR-103, miR-195, and miR-15b directly and specifically target the same site in the SALL4 3′UTR, as they have the same “seed” sequences. This does not affect the SALL4 mRNA stability, but rather suppresses SALL4 protein translation.

**Figure 6:**
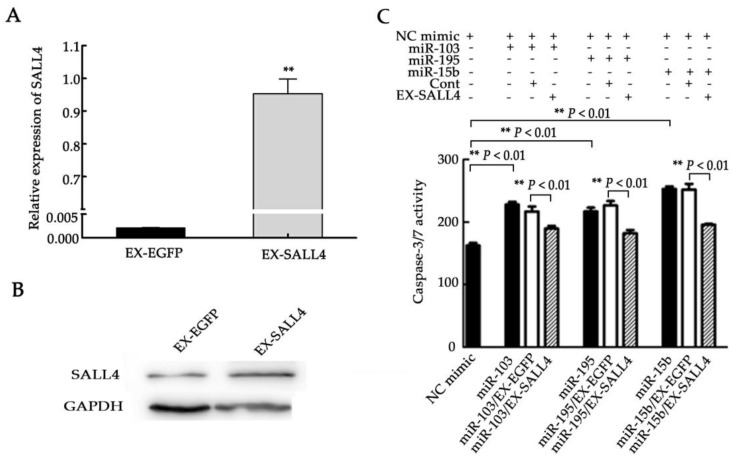
SALL4 overexpression counteracts the apoptosis-inductive effects of miR-103, miR-195, and miR-15b in glioma cells. (**A**,**B**) Lentiviral-mediated SALL4 overexpression was assessed by qRT-PCR (**A**) and the Western blot assay (**B**). After U251 cells were infected with EX-SALL4 lentiviral particles, the expression of SALL4 was significantly increased when compared to the control. (**C**) Caspase-3/7 activity was measured. SALL4 overexpression reversed the impact of miR-103, miR-195, or miR-15b on apoptosis induction in U251 cells. EX-EGFP is the control of the SALL4 overexpression group. Ex-SALL4: SALL4 overexpression group. NC mimic: negative control of miR-103, miR-195, and miR-15b mimics. Means ± SD (*n* = 3) are shown. **, *p <* 0.01.

To validate that miR-103, miR-195, and miR-15b promote cell apoptosis by directly downregulating SALL4, rescue experiments were performed with a SALL4 expression vector (EX-SALL4 lentiviral constructs) containing the SALL4 ORF without the 3′UTR. The qRT-PCR and Western blot analysis (Figure 6A,B) verified that SALL4 was overexpressed after infection with EX-SALL4 lentiviral particles (*p <* 0.01). Subsequently, SALL4-overexpressed U251 cells were transfected with miR-103, miR-195, or miR-15b mimics. The caspase-3/7 activity assay revealed that SALL4 upregulation partially rescued the apoptosis inductive effects of the miRNAs. U251 cells transfected with miR-103/Cont, miR-195/Cont, or miR-15b/Cont did not show significant changes in caspase-3/7 activity when compared to cells transfected with miR-103, miR-195, or miR-15b, respectively, since these cells preserved the gain-of-function of the three miRNAs (Figure 6C, *p <* 0.01). These results indicate that SALL4 is a common mediator of these miRNAs and induces cell apoptosis in U251 cells.

### 2.5. The Correlation between miR-103, miR-195, or miR-15b Expression, and SALL4 Level in Glioma Patients

**Figure 7:**
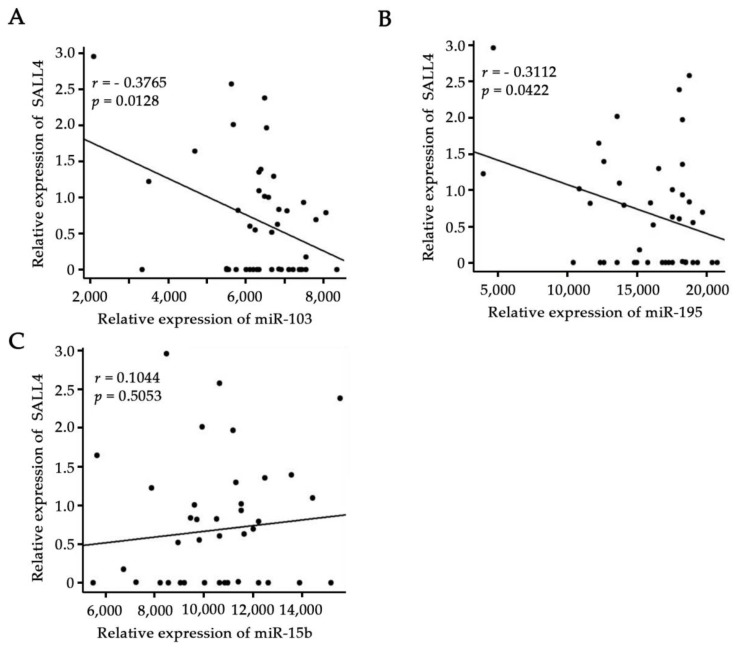
SALL4 expression showed a significant negative correlation with miR-103 or miR-195 levels in the clinical glioma samples (WHO IV, *n* = 43). (**A**) Downregulation of miR-103 was significantly associated with elevated SALL4 levels in these clinical glioma samples (*p <* 0.05). (**B**) Decreased miR-195 expression was significantly correlated with elevated SALL4 levels in the human glioma tissues (*p <* 0.05). (**C**) No significant correlation between miR-15b and SALL4 expression was observed (*p* = 0.5053).

The expression levels of miR-103, miR-195, miR-15b, and SALL4 from glioma samples (WHO IV, *n* = 43) were obtained from the Chinese Glioma Genome Atlas (CGGA, http://www.cgcg.org.cn/). The associations of SALL4 with these three microRNAs were analyzed in 43 glioblastoma patients with Spearman’s correlation analysis. The results showed an obvious inverse correlation between the expression of miR-103 or miR-195 and the SALL4 mRNA level (Figure 7A,B, *p <* 0.05). The correlation coefficients of miR-103/SALL4 and miR-195/SALL4, were −0.377 and −0.311, respectively. However, no significant correlation was observed between miR-15b and SALl4 expression in the same set of samples (Figure 7C, *p* = 0.5053). This may be due to the small sample size utilized in this study or other factors which need to be tested in the future. 

### 2.6. SALL4 Overexpression Promotes Cell Proliferation, Migration,and Invasion

**Figure 8:**
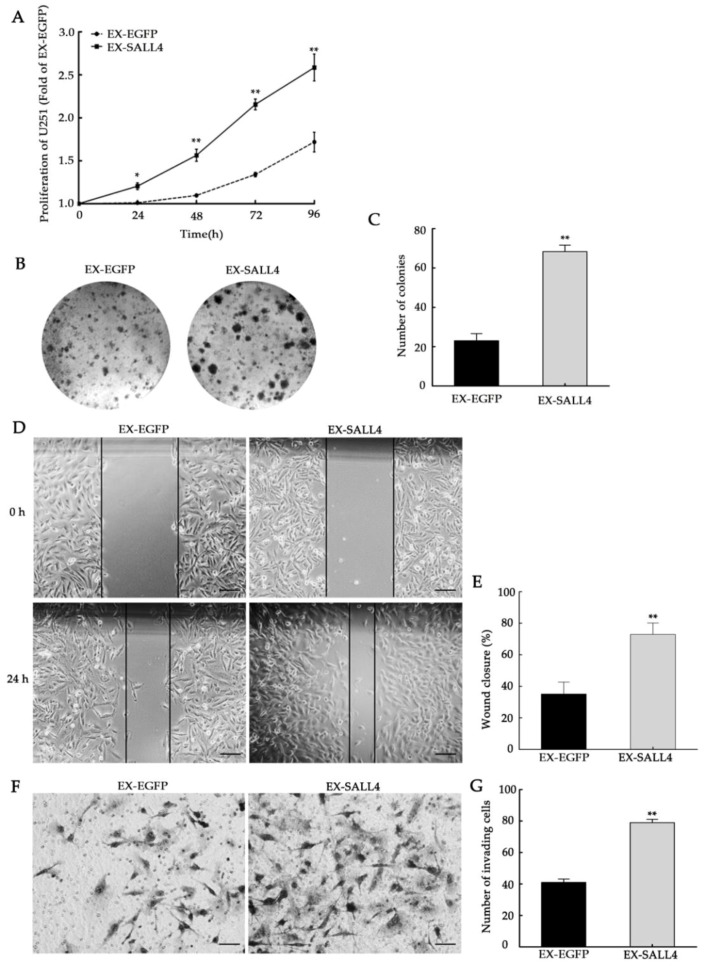
SALL4 overexpression promotes cell proliferation, migration, and invasion. (**A**) The proliferation of U251 cells after undergoing forced expression of SALL4 was significantly enhanced when compared with the control. (**B**,**C**) Colony formation assays in U251 cells. The colonies were imaged (**B**) and counted (**C**). (**D**,**E**) The migration of U251 cells after SALL4 overexpression was significantly increased when compared with the control. The percentage analysis of wound closure of (**D**) was shown in (**E**). (**F**,**G**) The invasion of U251 cells after SALL4 overexpression was significantly increased when compared with the control. The invasive cells were imaged (**F**) and counted (**G**) under representative microscopic fields. EX-EGFP is the control of the SALL4 overexpression group. Ex-SALL4: SALL4 overexpression group. Bar = 50μm. *, *p <* 0.05, **, *p <* 0.01 (*t*-test).

The role of SALL4 in stimulating cell proliferation has already been validated in our previous studies [11]. However, its role in regulating cell migration or invasion in glioma cells is currently unknown. To address this question, U251 cells were infected with EX-SALL4 lentiviral constructs that overexpressed SALL4. MTT and colony formation assay findings revealed that the overexpression of SALL4 markedly increased cell growth (Figure 8A–C, *p <* 0.01). Furthermore, the upregulation of SALL4 significantly promoted cell migration when compared to the scrambled control cells (Figure 8D,E, *p <* 0.01). Similarly, the overexpression of SALL4 triggered increased U251 cell invasion when compared with the control (Figure 8F,G, *p <* 0.01). In summary, these data show that SALL4 overexpression in U251 cells facilitates cell proliferation, migration, and invasion.

## 3. Discussion

miRNAs are small, noncoding RNAs that act as post-transcriptional regulators of mRNA expression via destabilization or translational repression and participate in the development, pathogenesis, and progression of cancer. Recent studies have revealed that several miRNAs, including miR-103, miR-195, and miR-15b, are directly involved in tumorigenesis and the metastasis of various types of cancer. In this study, we demonstrated, for the first time, that miR-103, miR-195, and miR-15b, which contain the same 5′ “seed” sequence, directly target SALL4 in glioma. Our data revealed that the three miRNAs play tumor suppressor roles in glioma cells by inhibiting cell proliferation, migration, and invasion, as well as by inducing cell apoptosis. 

Prior studies have shown that high expression of SALL4 is closely related to tumorigenesis and development [27,28,29,30,31]. Yang et al. reported that the reduction of SALL4 markedly diminishes the tumorigenicity of leukemic cells [27]. SALL4 has also been shown to promote the migration, invasion, and metastasis of lung cancer cells [28]. Pan et al. discovered that SALL4 activates long noncoding RNA DANCR in gastric cancer cells, and exerts its oncogenic activities through the activation of the β-catenin pathway [29]. More evidence showed that SALL4 is directly activated by TCF/LEF in the canonical Wnt signaling pathway [30]. The inhibition of SALL4 reduces tumorigenicity involving epithelial-mesenchymal transition via the Wnt/β-catenin pathway in esophageal squamous cell carcinoma [31]. In line with these reports, our studies indicated that SALL4 is linked to the promotion of glioma cell proliferation, migration, and invasion.

Recently, greater attention has been given to the regulation mechanisms of SALL4 by miRNAs. For instance, miR-181b, miR-219, miR-98, and miR-33b have been reported to target directly SALL4, and act astumor suppressors or metastasis suppressors in glioma, hepatocellular carcinoma, and breast cancer, respectively [13,15,32,33]. In the present study, SALL4 was revealed to be a confirmed target of miR-103, miR-195, and miR-15b, and was significantly downregulated by these three miRNAs in glioma cells. Furthermore, forced expression of SALL4 reversed the change in cell apoptosis induced by these miRNAs in U251 cells. Our study expanded on the epigenetic regulatory mechanism of SALL4 expression in glioma, highlighting the clinical importance of miR-103, miR-195, and miR-15b/SALL4 in the future treatment of glioma.

In recent years, many studies also indicated that miR-103, miR-195, and miR-15b function as tumor suppressors in the occurrence and development of cancers. miR-103 was reported to reduce cell proliferation and increase cell apoptosis by targeting the c-Myc activators known as c-Myb and DVL1 in hemopoietic tumor cells [22]. Fu et al. showed that miR-103 suppresses tumor cell proliferation by targeting PDCD10 in prostate cancer [19]. miR-195 and miR-15b belong to the miR-15 family. There have been accumulating reports showing the vital roles that miR-195 plays in suppressing tumorigenesis. For example, Yu et al. showed that miR-195 reduced tumorigenesis in non-small cell lung cancer by regulating cyclin D3 and survivin [34]. Other studies confirmed that miR-195 can target hTERT to inhibit the proliferation of melanoma cells [35]. Additionally, miR-15b is considered to be a negative regulatory factor in the growth of most human cancers. For example, Sun et al. demonstrated that miR-15b targeted cyclin D1 inhibits tumor growth and increases cell apoptosis in glioma [36]. Li et al. showed that miR-15b inhibits OIP5-mediated oncogenic signaling in human hepatocellular carcinoma [37]. However, miRNA expression shows high tissue and cell type specificity [38]. Although miR-103, miR-195, and miR-15b act as anti-oncogenes in most human cancers, some findings have revealed that they could function as oncogenes in other diseases. Zheng et al. reported that miR-103 enhances the proliferation of gastric cancer cells by targeting klf4 [39]. It was also found that miR-103 promotes neurite outgrowth and reduces cell apoptosis by targeting prostaglandin-endoperoxide synthase 2 (PTGS2) in cellular models of Alzheimer’s disease [40]. Recently, a few studies reported that miR-195 and miR-15b play roles that contribute to cell proliferation as oncogenes. For example, in U87 astrocytoma cells, miR-195 levels increased, which suggested an oncogenic role for miR-195 [24]. Work by Chen et al. verified that miR-15b targets RECK and enhances tumorigenesis in prostate cancer [26]. The above studies indicate that the exact functions of miR-103, miR-195, and miR-15b are different depending on the target and the cell type. In this study, our data were consistent with previous reports that miR-103, miR-195, and miR-15b function as tumor suppressors. miR-103, miR-195, and miR-15b were downregulated in glioma tissues. We verified that the overexpression of the three miRNAs suppressed proliferation, migration, and invasion and induced glioma cell apoptosis by targeting SALL4. In summary, it is tempting to speculate that the miR-103, miR-195, and miR-15b/SALL4 pathway might be an attractive target for a therapeutic intervention by inducing glioma cells to undergo apoptosis.

## 4. Materials and Methods

### 4.1. Ethics Statement

All subjects gave their informed consent for inclusion before they participated in the study. The study was conducted in accordance with the Declaration of Helsinki, and the protocol was approved by the Ethics Committee of the Fourth Affiliated Hospital at the Harbin Medical University.

### 4.2. Clinical Tissue Samples

A total of 47 fresh-frozen human glioma tissues and 13 normal brain tissues were obtained from the Fourth Affiliated Hospital of Harbin Medical University. All tumor tissues were excised from the cancer patients and diagnosed by using histopathology, in accordance with the World Health Organization (WHO) stage and grading system. The normal tissues were provided individuals who did not have cancer but had suffered a traumatic brain injury and undergone subsequent surgical resection. All tissue samples were immediately snap-frozen, and stored in liquid nitrogen until further use.

### 4.3. Cell Culture

U251 and MO59K human glioma cell lines were obtained from ATCC (Rockville, MD, USA), and U87 and SHG44 human glioma cell lines were purchased from the China Academia Sinica Cell Repository, Shanghai, China. All cell lines were cultured in Dulbecco’s modified Eagle’s medium (DMEM, Gibco, Carlsbad, CA, USA) containing 10% of fetal bovine serum (FBS, Gibco, Carlsbad, CA, USA). The cells were maintained at 37°C with 95% humidity and 5% CO_2_.

### 4.4. Oligonucleotides and Cell Transfection

The miR-103, miR-195, or miR-15b mimics and inhibitors were RNA duplexes. The oligonucleotides were chemically synthesized by RIBOBIO (Guangzhou, China). The negative control RNA duplex was not homologous to any human genome sequence. In accordance with the manufacturer’s instructions, glioma cells were transfected with 50 nM miRNA mimics or 100 nM inhibitors by using the Lipofectamine 2000 reagent (Invitrogen, Carlsbad, CA, USA). Following incubation at 37°C and 5% CO_2_ for 6 h, the transfection mixture was replaced with DMEM with 10% FBS. At 48 h after transfection, the total RNAs or proteins were isolated and used for qRT-PCR analysis or Western blotting assays, respectively.

### 4.5. Lentiviral Construct Transduction

For SALL4 overexpression, lentiviral particles with EX-SALL4 constructs were packaged in 293T cells by using the Lenti-Pac HIV Expression Packaging Kit (HPK-LvTR-20), in accordance with the manufacturer’s instructions (Genecopoeia, Carlsbad, CA, USA). U251 cells were incubated with viral particles and mixed with 8 μg/mL of polybrene (Sigma-Aldrich, St. Louis, MO, USA) overnight. Subsequently, the culture medium was then removed and replaced with Dulbecco’s modified Eagle’s medium (Gibco, Carlsbad, CA, USA) containing 10% FBS (Gibco, Carlsbad, CA, USA). The cells were harvested after lentiviral construct transduction for 48 h in total.

### 4.6. Real-Time Quantitative Reverse-Transcription (RT)-PCR 

For mRNA expression detection, total RNA was extracted from tissues and cell lines with the TRIzol reagent (Thermo Fisher Scientific, Inc., Carlsbad CA, USA). RNA was converted into cDNA with a PrimeScript 1st Strand cDNA Synthesis kit (Takara Bio., Inc., Tokyo, Japan). The SALL4 and GAPDH transcripts were amplified in the presence of SYBR Green (Takara, Tokyo, Japan) with the Applied Biosystems 7500 Real-Time PCR System. For quantification, the SALL4 expression levels were normalized to GAPDH.

The primers for SALL4 were as follows: forward, 5′-TGCAGCAGTTGGTGGAGAAC-3′; and reverse, 5′-TCGGTGGCAAATGAGACATTC-3′. The primers for the internal control gene GADPH were as follows: forward, 5′-CTGGGCTACACTGAGCACC-3′; and reverse, 5′-AAGTGGTCGTTGAGGGCAATG-3′.

For miR-103, miR-195, and miR-15b expression detection, the total RNA was isolated with a miRNA isolation kit (Applied Biosystems, Carlsbad, CA, USA), as suggested by the manufacturer, and used for reverse transcription with the microRNA Reverse Transcription Kit (Applied Biosystems, USA). The probes for miR-103, miR-195, miR-15b, and U6 small nuclear RNA detection were purchased from Life Technologies corporation (Gaithersburg, MD, USA). RNA expression of mature miR-103, miR-195, and miR-15b was measured with TaqMan miRNA Assays (Applied Biosystems) in accordance with the manufacturer’s instructions. The expression levels of the three miRNAs were normalized to the expression level of the endogenous U6 small nuclear RNA with the 2^−ΔΔCt^ relative quantitative method. 

### 4.7. Western Blot

Protein extracts from cells were isolated in the RIPA lysis buffer (25mM Tris-HCl (pH 7.4), 10% (*v/v*) glycerol, 150mM NaCl, 2mM EDTA) with cocktail protease inhibitors (Thermo Fisher Scientific, Inc., Carlsbad CA, USA). Then, protein concentrations were determined with a Thermo Scientific Pierce BCA Protein Assay Kit and denatured in a 1 × Laemmli’s gel loading buffer. The proteins were separated by a 12% SDS-PAGE, which was followed by immunoblotting onto a polyvinylidene difluoride membrane (Thermo Fisher Scientific, Inc., Carlsbad, CA, USA). The Western blot analyses were performed with antibodies specific to SALL4 (1:800 dilution, Abcam, Cambridge, MA, USA) and β-tubulin (1:1000 dilution, Santa Cruz, Dallas, TX, USA). An ECL kit (Pierce Chemical, Rockford, IL, USA) was used to perform chemiluminescence detection,in accordance with the manufacturer’s instructions. The relative protein expression was represented by the density ratio versus the β-tubulin.

### 4.8. Hematoxylin and Eosin (H&E) Staining

Glioma tissues or normal brain tissues were fixed in 4% paraformaldehyde (PFA) in PBS. These tissues were embedded in paraffin and sectioned to 4 or 5 μm sections, followed by deparaffinization with xylene for re-hydration in absolute alcohol, 95% alcohol, and 70% alcohol. Then, they were stained in Harris hematoxylin solution, and differentiated in 1% acid alcohol. These tissue sections were stained blue in 0.2% ammonia water, rinsed in 95% alcohol, and counterstained in an eosin–phloxine solution. Subsequently, they were dehydrated with 95% alcohol and absolute alcohol and cleared in xylene. Lastly, they were mounted with a xylene-based mounting medium.

### 4.9. Cell Proliferation Assays

Cell proliferation was measured with an MTT assay. The cells were seeded in a 24-well cell culture plate. Twenty-four hours after transfection with miR-103, miR-195, miR-15b mimics or inhibitors, they were seeded into 96-well culture plates and incubated for 24, 48, or 72 h. Subsequently, 50 µL of 3-(4,5-dimethylthiazol-2-yl)-2,5-diphenyltetrazolium bromide (MTT) solution (5 mg/mL, KeyGEN, Biotech, Nanjing, China) was added to each well on each of the 3 consecutive days after transfection. The cells were then incubated at 37°C for 4 h. The supernatant was discarded and 200 µL of DMSO was added to each well to solubilize the crystals. The optical density (OD) was measured at 490 nm with a microplate spectrophotometer (Infinite M200, TECAN, Crailsheim, Germany).

### 4.10. Colony Formation Assays

Colony formation assays were performed to assess the proliferation ability of transfected U251 cells. Briefly, the culture medium was removed after transfection for 24 h, and the cells (4 × 10^2^/well) were suspended in 2 mL of DMEM medium containing 10% FBS in 6-well plates. Each assay was performed in triplicate. After 3 weeks of incubation at 37 °C in a 5% CO_2_ incubator, the cell colonies were fixed with methyl alcohol for 10 min, and stained with a 1% crystal violet solution for 5 min. Images of the colonies were then captured, and the number of colonies (≥100 µm in diameter) was counted by using Image-Pro Plus software (6.0, Media Cybernetics, Inc., Rockville, MD, USA).

### 4.11. Transwell Migration and Invasion Assays

Cell migration and invasion were assessed with Transwell assays. For the cell migration assay, transfected cells undergoing different treatments were plated at a density of 2 × 10^4^ cells per well in the upper chambers (8.0 μm pore size with polycarbonate membrane, BD Biosciences, Cowley, UK) and were filled with serum-free medium. The lower chamber was filled with DMEM medium containing 10% FBS. For the invasion assays, the invasion of cells through the matrigel was determined by using a 24-well matrigel invasion chamber with a pore size of 8 μm (BD Biosciences, Cowley, UK). Cells with different transfections were suspended in medium without serum at a concentration of 2 × 10^4^ cells/well, and immediately placed into the upper chambers. Subsequently, the lower chamber was filled with complete medium (DMEM medium containing 10% FBS). 

The cells were incubated at 37 °C in a 5% CO_2_ incubator for 12 h (migration assay) or 24 h (invasion assay). Following incubation, the non-migrating or non-invading cells were removed from the upper surface of the membrane by wiping with cotton-tipped swabs. The cells on the lower surface of the membrane were fixed in 4% paraformaldehyde for 15 min and stained with a 1% crystal violet solution for 5 min. Five fields of adherent cells were randomly captured with a microscope (Olympus, Tokyo, Japan) at 10× magnification. The migrating/invading cells were counted by using Image-Pro Plus software. 

### 4.12. Wound Healing Assay

The migration of SALL4-overexpressing cells was measured with the wound healing assay in 6-well plates. Wounds of approximately 1 mm width were created with a plastic scriber in each well after the cultured cells became fully confluent. After scratching, the cells were cultured in FBS-free DMEM medium with mitomycin C (25 μg/mL) for 30 min. The medium was then replaced with DMEM medium containing 10% FBS, and the cells were cultured at 37 °C in a 5% CO_2_ incubator. Microscopic pictures of the cultures were taken at 0, 24, 48, and 72 h.

### 4.13. Apoptosis Assay

Cell apoptosis was quantified 72 h after the different transfection treatments with annexin V and propidium iodide (PI) double staining and caspase-3/7 activity assays. Annexin V-FITC and PI double staining with the Apoptosis Detection Kit (BD Biosciences, San Jose, CA, USA) was used to evaluate the percentages of apoptotic cells, in accordance with the manufacturer’s protocol and analyzed by flow cytometry (BD FACSCalibur, BD Biosciences, San Jose, CA, USA). Caspase-3/7 activity was measured in 96-well microassay plates with a Multi-Detection Microplate Reader (Bio-Tek, Winooski, VT, USA), which used the Caspase-Glo 3/7 reagent, based on the manufacturer’s protocol (Promega, Madison, WI, USA). The assessment of apoptosis was repeated in triplicate.

### 4.14. Luciferase Reporter Assay

The luciferase reporter assay was conducted to confirm the association between miRNA (miR-103, miR-195, and miR-15b) and its potential target, SALL4. Cells maintained in 24-well plates were co-transfected with SALL4 3′UTR pMIR-REPORT luciferase plasmids (with either wild-type or mutant binding sites of miR-103, miR-195, or miR-15b), *Renilla* luciferase plasmids and mimics (miR-103, miR-195 or miR-15b), or inhibitors using the Lipofectamine 2000 reagent (Invitrogen, Carlsbad, CA, USA). The firefly and *Renilla* luciferase activities were measured 48 h after transfection using the Dual Luciferase Reporter Assay Kit (Promega, Madison, WI, USA). The firefly luciferase activity was normalized to the *Renilla* luciferase activity.

### 4.15. MiRNA Target Site Prediction

The prediction of miRNA target sites was performed by using the TargetScan (http://www.targetscan.org) and the miRBase Targets (http://www.mirbase.org).

### 4.16. Statistical Analysis

Excel software and GraphPad Prism 5 were used for the statistical analysis. The histograms represent the mean values and the bars indicate the standard deviation of the mean. The statistical significance of the results was determined by Student’s *t*-tests with data considered significant when *p <* 0.05. The degree of the linear relationship between SALL4 and miR-103, miR-195, or miR-15b expression was calculated in 43 glioblastoma specimens from the Chinese Glioma Genome Atlas (CGGA, http://www.cgcg.org.cn/) by using the Spearman’s rank correlation. 

## 5. Conclusions

Our results revealed critical roles for miR-103, miR-195, and miR-15b as tumor suppressors in glioma through the repression of SALL4 translation. However, our findings were derived from glioma cell lines, and cannot replace an accurate study in a clinical context. More functional in vivo experiments are needed to validate the effects of miR-103, miR-195, and miR-15b, and how they regulate the expression of other target genes in glioma, as well as in the broad scope of other cancers.

## Supplementary Materials

The following are available online at http://www.mdpi.com/1420-3049/23/11/2938/s1, Figure S1: The database predicted that miR-103 (A) miR-195 (B) and miR-15b (C) with the same 5′ “seed” sequence matched the same site in the SALL4 mRNA 3′UTR. Figure S2: Representative HE staining images from 13 normal brain tissue samples and 47 different grading glioma samples. Figure S3: Transfection efficiency of miR-103, miR-195, and miR-15b mimics. Figure S4: Transfection efficiency of miR-103, miR-195, and miR-15b inhibitors. Figure S5: The expression of SALL4 mRNA in glioma cells transfected with mimics or inhibitors of miR-103, miR-195, or miR-15b.
