# Supplementary figures and images for: miR-103/miR-195/miR-15b Regulate SALL4 and Inhibit Proliferation and Migration in Glioma
Source: Molecules. 2018 Nov 10;23(11):2938. doi: 10.3390/molecules23112938 (PMC6278493; doi:10.3390/molecules23112938)

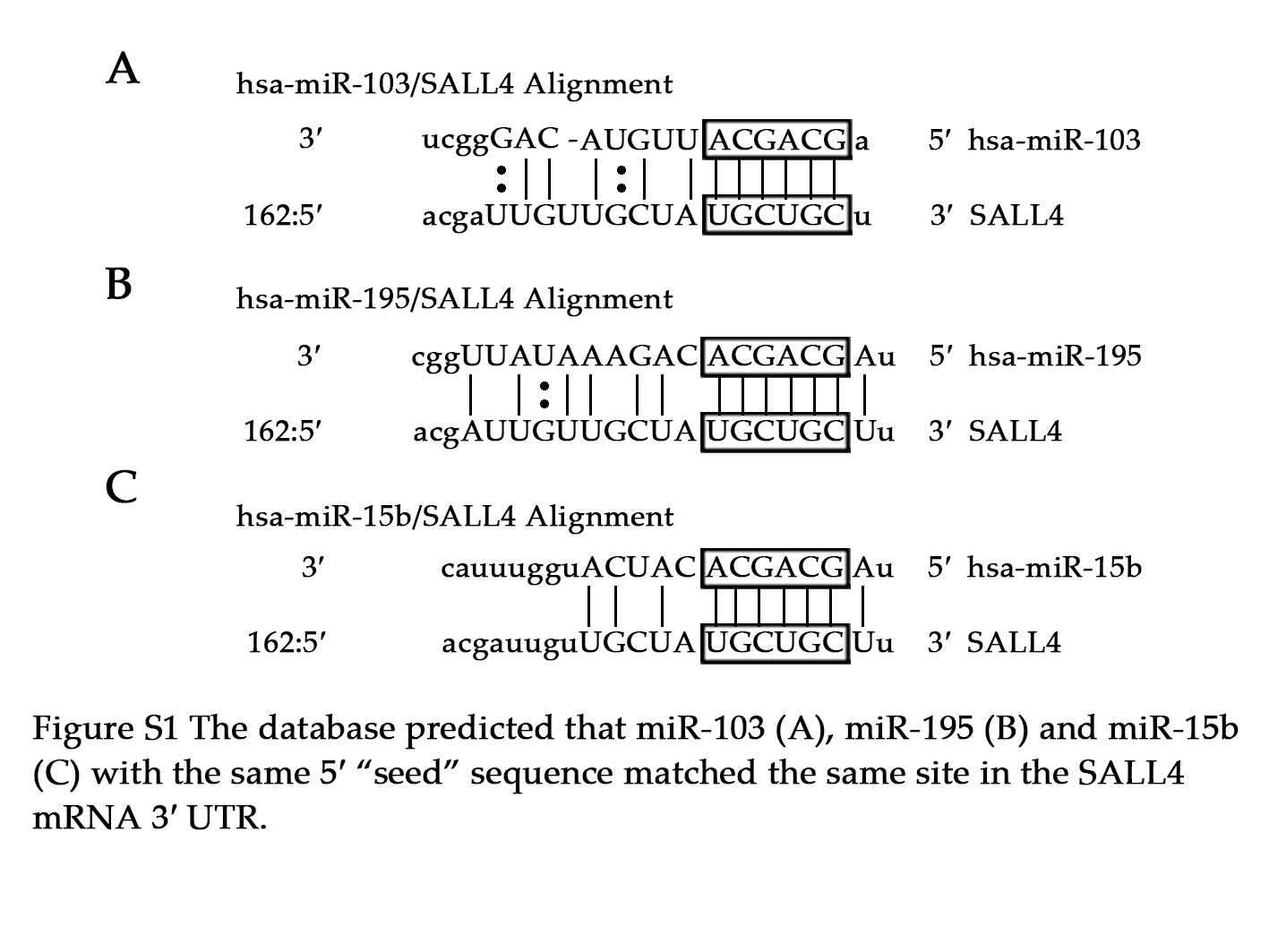

Supplement: Supplementary file 1 [file molecules-23-02938-s001.zip › supplement figure3/图S1.jpg]

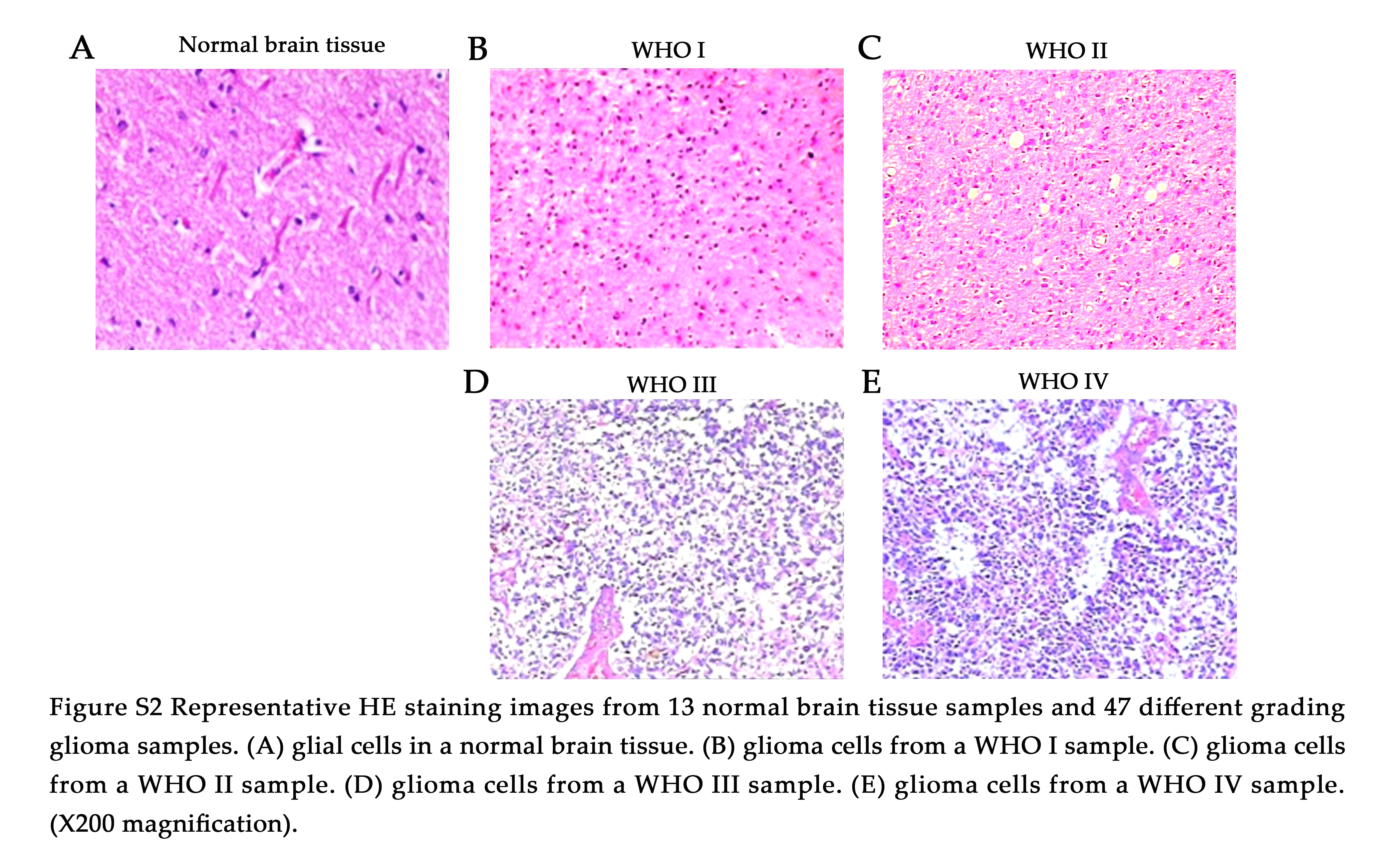

Supplement: Supplementary file 1 [file molecules-23-02938-s001.zip › supplement figure3/图S2.jpg]

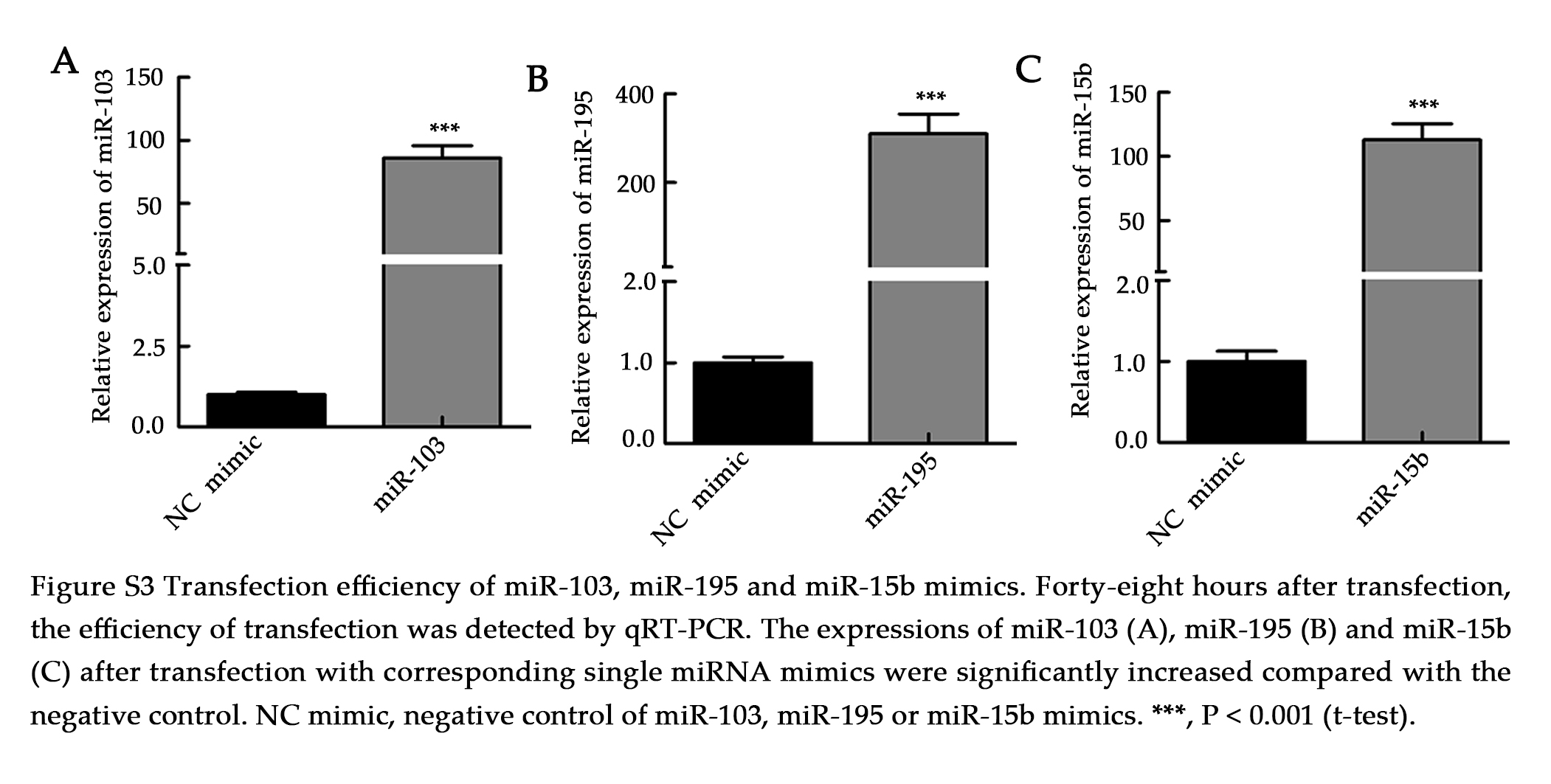

Supplement: Supplementary file 1 [file molecules-23-02938-s001.zip › supplement figure3/图S3.jpg]

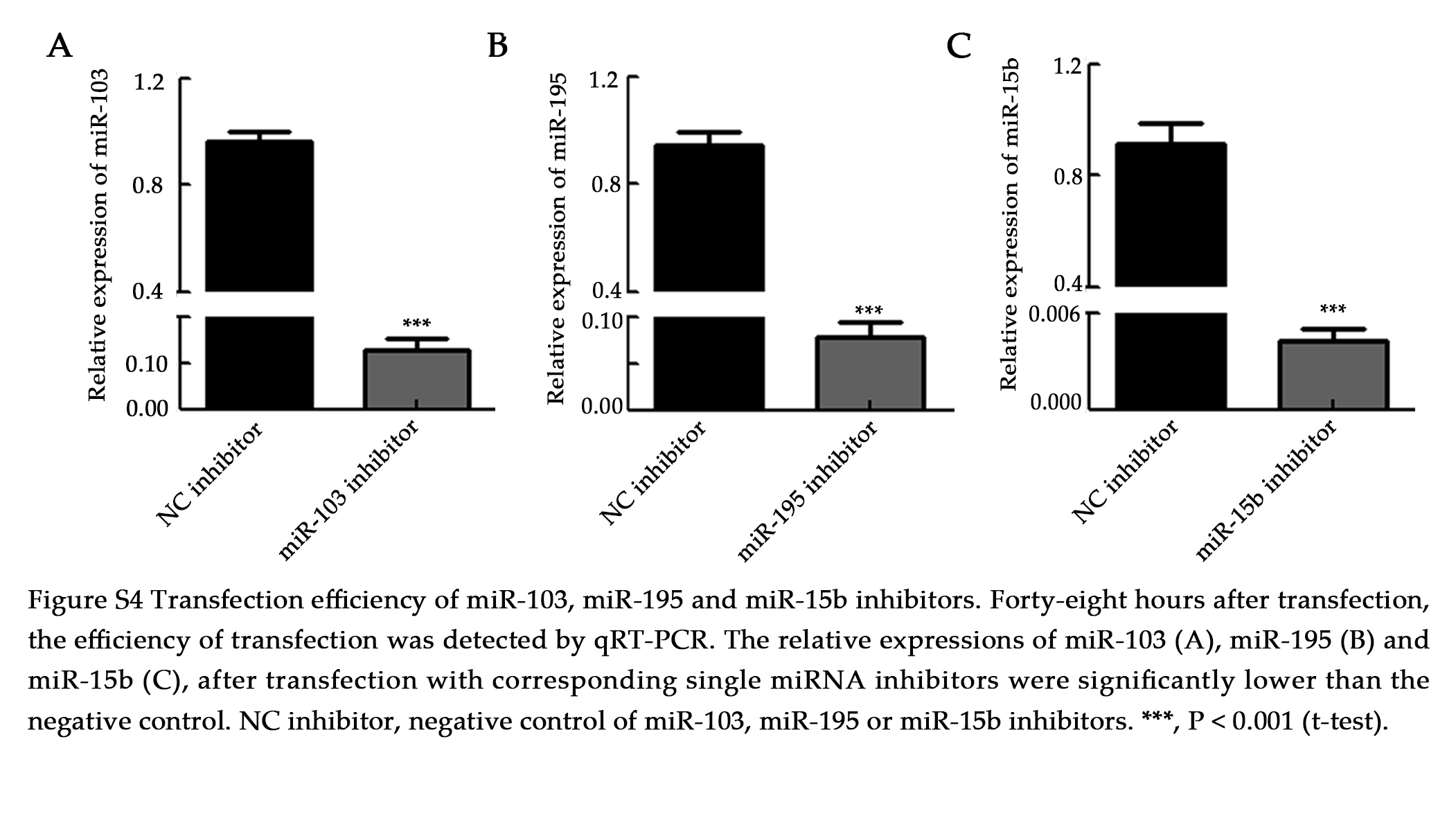

Supplement: Supplementary file 1 [file molecules-23-02938-s001.zip › supplement figure3/图S4.jpg]

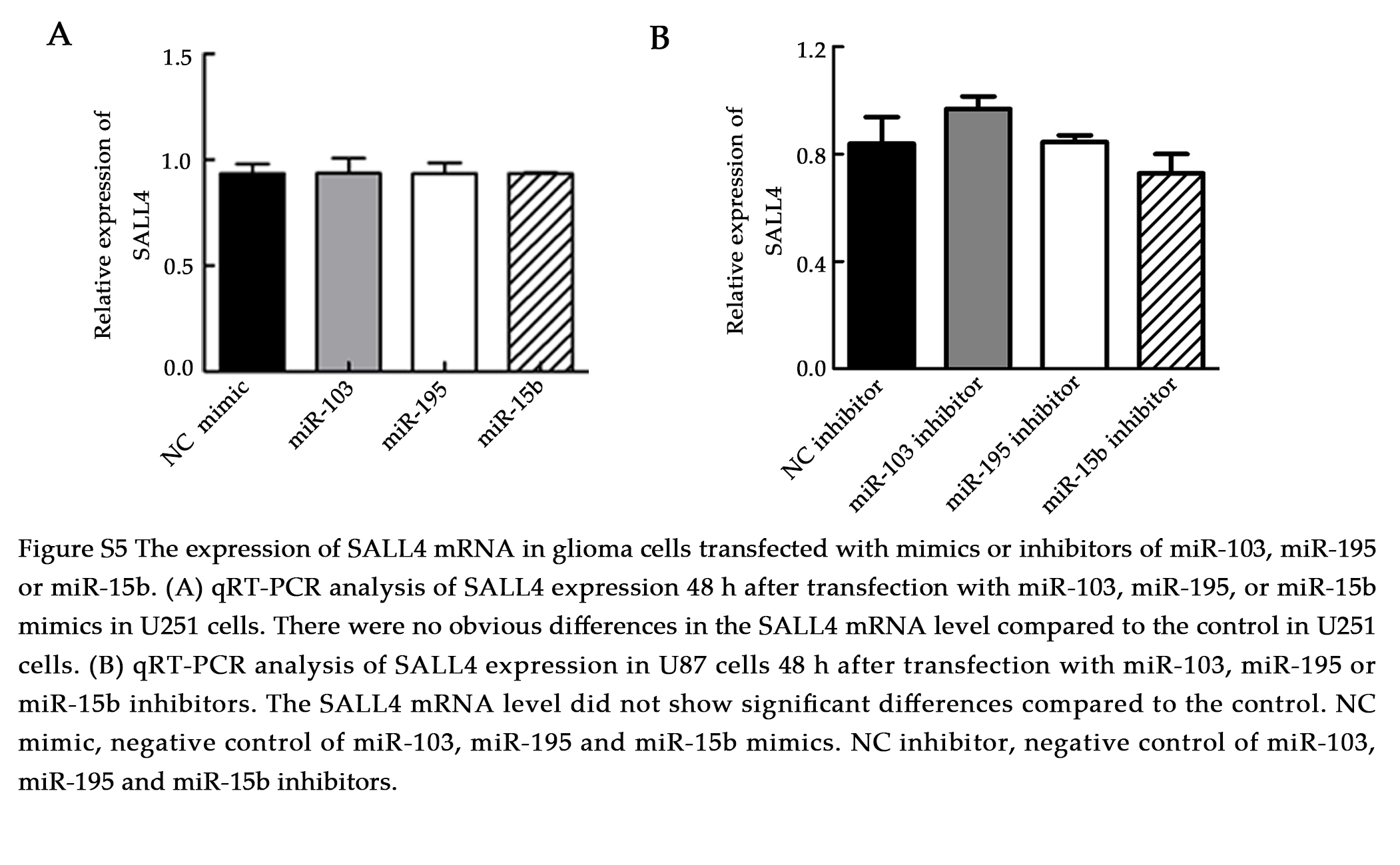

Supplement: Supplementary file 1 [file molecules-23-02938-s001.zip › supplement figure3/图S5.jpg]
